# Supplementary figures and images for: Examining the burden and relationship between stunting and wasting among Timor-Leste under five rural children
Source: PLoS One. 2024 Oct 25;19(10):e0312433. doi: 10.1371/journal.pone.0312433 (PMC11508072; doi:10.1371/journal.pone.0312433)

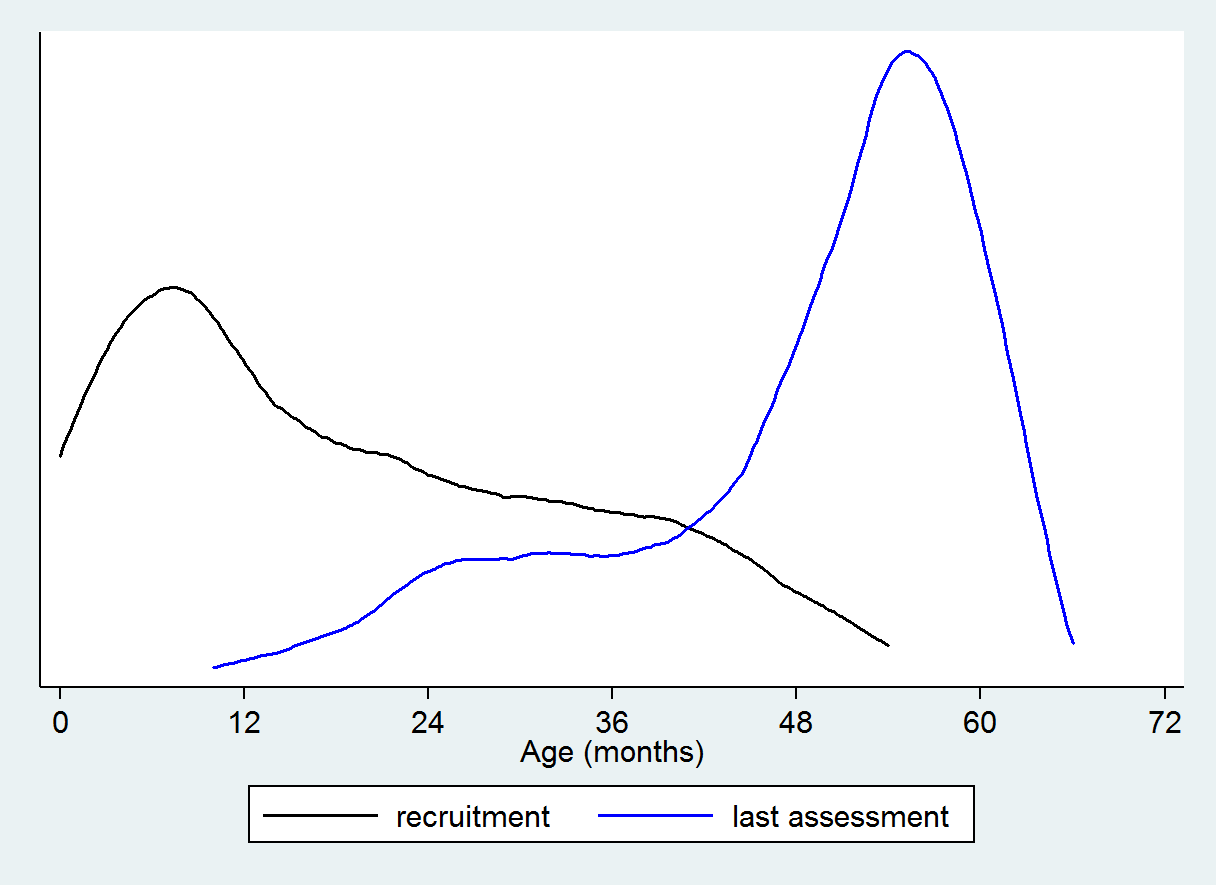

Supplement: S1 Fig — (TIF) [file pone.0312433.s001.tif]
