# Supplementary material for: Examining the burden and relationship between stunting and wasting among Timor-Leste under five rural children
Source: PLoS One. 2024 Oct 25;19(10):e0312433. doi: 10.1371/journal.pone.0312433 (PMC11508072; doi:10.1371/journal.pone.0312433)
Supplement: Attachment — Submitted filename: Responses to Reviewers.docx [file pone.0312433.s002.docx]

PONE-D-23-40750

Examining the burden and relationship between stunting and wasting among Timor-Leste under five rural children.

Dear editors and reviewers,

Thanks for your investment in improving our manuscript. Please, find below the point-by-point responses to your comments and queries.

Journal requirements:

When submitting your revision, we need you to address these additional requirements.

1. Please ensure that your manuscript meets PLOS ONE's style requirements, including those for file naming. The PLOS ONE style templates can be found at

<https://journals.plos.org/plosone/s/file?id=wjVg/PLOSOne_formatting_sample_main_body.pdf> and

<https://journals.plos.org/plosone/s/file?id=ba62/PLOSOne_formatting_sample_title_authors_affiliations.pdf>

Thank you for this recommendation. We have made all the verification to ensure that we fully adhere to PLOS ONE’s requirements.

2. We note that the grant information you provided in the ‘Funding Information’ and ‘Financial Disclosure’ sections do not match.

When you resubmit, please ensure that you provide the correct grant numbers for the awards you received for your study in the ‘Funding Information’ section.

Thanks for spotting this. Changes have been made to take into account your comments 2 and 3.

3. Thank you for stating the following in the Acknowledgments Section of your manuscript:

“The authors would like to thank Roland Kupka and Paul Binns for their insightful comments that improved the manuscript. We also thank the University of Western Australia research team for sharing the dataset with us. Thanks also to the UNICEF Timor Leste, UNICEF East Asia and the Pacific Regional office (EAPRO) and Action Against Hunger teams for the administrative and technical support provided throughout the study implementation. The original study was funded by the Australian Research Council (Grant DP 120101588), the School of Anatomy, Physiology, and Human Biology at the University of Western Australia, and the Timor-Leste UNICEF office. The funders played no role in the design of this secondary data analysis study and the interpretation of the results”

We note that you have provided funding information that is currently declared in your Funding Statement. However, funding information should not appear in the Acknowledgments section or other areas of your manuscript. We will only publish funding information present in the Funding Statement section of the online submission form.

Please remove any funding-related text from the manuscript and let us know how you would like to update your Funding Statement. Currently, your Funding Statement reads as follows:

“DSJ and PS received funding for the data collection for the original study from the Australian Research Council. The School of Anatomy, Physiology, and Human Biology at the University of Western Australia and UNICEF Timor-Leste.”

Please include your amended statements within your cover letter; we will change the online submission form on your behalf.

Thanks this is understood and done.

Revised text

The original study was funded by the Australian Research Council (Grant DP 120101588), the School of Anatomy, Physiology, and Human Biology at the University of Western Australia, and the Timor-Leste UNICEF office. This particular study (secondary data analysis and paper writing) was funded by the European Union through the UNICEF Timor-Lester office. The funders played no role in the design of this secondary data analysis and the interpretation of the findings

4. When completing the data availability statement of the submission form, you indicated that you will make your data available on acceptance. We strongly recommend all authors decide on a data sharing plan before acceptance, as the process can be lengthy and hold up publication timelines. Please note that, though access restrictions are acceptable now, your entire data will need to be made freely accessible if your manuscript is accepted for publication. This policy applies to all data except where public deposition would breach compliance with the protocol approved by your research ethics board. If you are unable to adhere to our open data policy, please kindly revise your statement to explain your reasoning and we will seek the editor's input on an exemption. Please be assured that, once you have provided your new statement, the assessment of your exemption will not hold up the peer review process.

Thanks. The statement has been modified. The link to the datasets is now provided.

5. PLOS requires an ORCID iD for the corresponding author in Editorial Manager on papers submitted after December 6th, 2016. Please ensure that you have an ORCID iD and that it is validated in Editorial Manager. To do this, go to ‘Update my Information’ (in the upper left-hand corner of the main menu), and click on the Fetch/Validate link next to the ORCID field. This will take you to the ORCID site and allow you to create a new iD or authenticate a pre-existing iD in Editorial Manager. Please see the following video for instructions on linking an ORCID iD to your Editorial Manager account: <https://www.youtube.com/watch?v=_xcclfuvtxQ>

We have faced challenges linking the paper to our ORCID because the ORCID was registered with an email no longer active and the system is not accepting the updating of the email...

Reviewers' comments:

Reviewer #1:

Dear Authors

Thank you for this manuscript. I have minor issues to be considered.
1. As I understand you used secondary data from previous surveys, in the data collection section, you describe how the surveys were conducted. I think it is important that you describe how you conducted data collection for this paper, how did you select the surveys, who did that, where did you get the sources, were they in your possession or did you have to acquire from government institutions?

Thank you for this comment. We have revised the sub-section study design (page 6, lines 130-132; and 135-1138) and the sub-section data collection and procedures (pages 7, lines 162-169) to address it.

2. Adequate nutrition during infancy and early childhood is essential to ensure the growth, health, and development of children to their full potential. Stunting and wasting is influenced by nutrition. How was nutrition during infancy and childhood in the surveys controlled? In your statistical analysis to determine the relationship between stunting and wasting did you use feeding in the regression analysis?

Thank you for this comment. We agree with you that nutrition feeding during infancy and early childhood indicators should have been considered for inclusion in our regression analyses. Unfortunately, the information was not available for a significant proportion of children of our sample. We have listed this fact as one of the weakness of our analyses. We have slightly revised the paragraph on strengths and weaknesses to specifically mention feeding history and practices (page 33, lines 728-730).

3. Since this is not a new subject, state clearly the new knowledge you are bringing to literature.

Thank you for this suggestion. We have revised the conclusion section in response to this comment (page 34, lines 749-755).

Reviewer #2:

Dear Authors, thank you for the chance to me for review the manuscript entitled: Examining the burden and relationship between stunting and wasting among Timor-Leste under five rural children. This manuscript is very important to solve public health problem, particularly for under-five children with stunting issues in low-middle income countries. However, before to publish this manuscript, there are any issues that should add and clarify to improve this manuscript.

1. In title the authors mentioned about rural areas, please describe some issues about fulfilling nutrition for under-five children in the rural areas, especially in low-middle income countries.

Thank you for the suggestion. We have added some mention on this in the introduction (page 4, lines 66-69).

2. This research was conducted in Timor-Leste, please describe any program that government done to solve this problem. Therefore, we know the importance this study should be done.

Thank you for your suggestion that we have implemented. A full paragraph has been added in the introduction section to provide the requested information (pages 4-5, lines 70-101).

3. In the method. Please describe more about Timor-Leste. How many public health centers on there and how they develop program to solve stunting. How many areas that included in this study and how was done to collect the data in this study.

Thank you again for this suggestion. We have implemented the suggestion by added the proposed information in the relevant sections and sub-sections of the manuscript including introduction (page 4-5, line 70-101), setting (page 6-7, lines 143-155), and data collection sub-section of the methods (page7, lines 162-169).

4. The results are well-written and reflected the study.

Thank you very much for this positive feedback.

5. Discussion. The authors said that they are missed about stunting and wasting in Timor Leste. Please describe how the healthcare providers on there to measure about height and weight. We think that measurement and recorded the assessment in public health setting is important issues. Sometimes, they have limited equipment or missed to record in growth chart, etc.... please describe more about the field observation on there and discussed.

Thank you for this comment but we think that this comment is highlighting an issue different to the point we are making in our paper. We agree with the reviewer that it is challenging to obtain accurate anthropometric measurements during routine activities in most low and middle income countries and this can affect the level of identification of cases but our point is that cases identified are addressed by different programs. In fact, our aim is to highlight the issues with current policy and programmatic environment.

With regards to the potential effects of errors of measurements on presented results, as mentioned in the methods section, for this study, the measurements were done in research context by well-trained and skilled research assistants and not in routine health services context.

6. Conclusion is well-written and answered the objective of study.

Thank you for this positive feedback.

7. Reference. Please add some references from Indonesia and Papua Nugini to compare and contract the findings, because this is some areas with stunting cases.
We have added two references on prevalence of undernutrition in Indonesia and Papua New Guinea.
